# Systematic Review of Stem-Cell-Based Therapy of Burn Wounds: Lessons Learned from Animal and Clinical Studies

**DOI:** 10.3390/cells9122545

**Published:** 2020-11-26

**Authors:** Josefine Lin Henriksen, Nana Brandborg Sørensen, Trine Fink, Vladimir Zachar, Simone Riis Porsborg

**Affiliations:** 1 Department of Health Science and Technology, Aalborg University, Fredrik Bajers Vej 7, 9220 Aalborg, Denmark; lin.henriksen@rn.dk (J.L.H.); nana.s@rn.dk (N.B.S.); 2 Regenerative Medicine Group, Department of Health Science and Technology, Aalborg University, Fredrik Bajers Vej 3B, 9220 Aalborg, Denmark; trinef@hst.aau.dk (T.F.); vlaz@hst.aau.dk (V.Z.)

**Keywords:** burn wound, mesenchymal stem cell, stem-cell-based treatment, adipose-derived stem cells, ASC, MSC

## Abstract

Treatment of severe burn wounds presents a daunting medical challenge, and novel approaches promoting healing and reducing scarring are highly desirable. The application of mesenchymal stem/stromal cells (MSCs) has been suggested as a novel treatment. In this paper, we present systematic reviews of pre-clinical and clinical studies of MSC therapy for second- or third-degree thermal burn wounds. Following the Preferred Reporting Items for Systematic Reviews and Meta-Analysis (PRISMA) guidelines, the PubMed and Embase databases were searched, and interventional studies of MSC therapy using rodent models (21 studies) or human burn patients (three studies) were included in the pre-clinical and clinical reviews, respectively, where both overall outcome and wound-healing-phase-specific methodologies and effects were assessed. The pre-clinical studies demonstrated a promising effect of the application of MSCs on several wound healing phases. The clinical studies also suggested that the MSC treatment was beneficial, particularly in the remodeling phase. However, the limited number of studies, their lack of homogeneity in study design, relatively high risk of bias, lack of reporting on mode of action (MOA), and discontinuity of evidence restrict the strength of these findings. This comprehensive review presents an overview of available methodologies to assess the MOA of MSC treatment for distinct wound healing phases. Furthermore, it includes a set of recommendations for the design of high-quality clinical studies that can determine the efficacy of MSCs as a therapy for burn wounds.

## 1. Introduction

Since the destruction of 15% of the total body skin surface is sufficient to result in a life-threatening condition, the treatment of severe burn wounds presents a serious medical challenge. Most significantly, burn wounds are associated with hypovolemic shock, but they can also lead to other dysregulations, such as hypermetabolism or immunosuppression [1]. Long-term consequences may then involve scarring, soft-tissue deformities, and fibrosis. These are frequently associated with overall loss of mobility and pain [2].

The burn wounds are distinctive by a high degree of vascular permeability, but their healing follows a general paradigm common to all wounds. The reparative processes are initiated with an inflammatory response, which is histologically marked by neutrophil and macrophage infiltration. This phase is followed by a proliferative phase, where new granulation tissue is formed along with new blood vessels, and the surface is re-epithelialized. During the final phase of maturation/remodeling, the extracellular matrix (ECM) is remodeled and collagen type III is replaced with collagen type I. It should be noted that the proliferative phase is impaired with increasing depth of injury, and this has adverse consequences for the re-epithelialization and remodeling processes. Consequently, the scarring after severe burns evolves over the years and has a tendency towards hypertrophy [3].

Thus, there is a need for developing approaches that promote healing, reduce scarring, and restore normal skin function. The application of MSCs has been suggested as a novel therapy for burn wounds [4], as these cells have been shown to influence a number of factors associated with the different phases of wound healing. In terms of the effect on the inflammatory phase, MSCs can interact with cells of the immune system either through secretion of soluble factors or through direct cell–cell contact. This immunomodulatory interaction with T and B cells, natural killer (NK) cells, neutrophils, macrophages, and dendritic cells supports a more anti-inflammatory environment [5,6]. The MSCs may also have an impact on the proliferative phase, as we and others have shown that these cells secrete the pro-angiogenic vascular endothelial growth factor (VEGF) [7,8,9,10], but other factors with pro-trophic and anti-apoptotic effects appear to play a role as well [11]. The functional significance of the MSC secretome for wound healing has been demonstrated in vitro in dermal fibroblast-, keratinocyte-, and endothelial-cell-based scratch assays [12,13,14]. Finally, regarding the impact on the remodeling phase, in vitro studies indicate that MSCs produce a number of factors relevant to the composition and regulation of the ECM [15,16].

The MSCs are found in several tissues, such as the adipose tissue, bone marrow, amniotic membrane, umbilical cord, and dental pulp. For MSCs derived from adipose tissue (ASCs) and bone marrow (BM-MSCs), protocols for isolation and expansion of the clinical-grade stem cells have already been developed, and the clinical utility in at least the selected indications, such as perianal fistulas or ischemic heart disease, has been confirmed [17,18,19,20,21]. Additionally, clinical studies on diabetic foot ulcers [22] and venous ulcers [23] are suggestive that MSCs might also be beneficial in the case of burn wounds; nevertheless, a comprehensive understanding of MSCs’ significance in this scenario is yet to be obtained.

The aim of this article is to critically access the clinical potential of MSCs for burn wounds. This is done through two systematic reviews analyzing the current state of knowledge. The first review is centered on stem cell treatment of burn wounds in rodent models. It attempts to clarify the molecular and cellular effects of stem cell treatment on the different phases of wound healing. It also strives to identify methodologies that would be easy to translate into the clinical studies. The second review deals with clinical studies in order to evaluate the efficacy of MSCs for treatment of severe burn wounds and to clarify the advancement in the process of clinical translation of the use of stem cells for burn wounds. The animal-based review is methodologically heavy, but it might be beneficial for the translation. Furthermore, it might aid the design and increase the quality of future resource-intensive clinical trials. These are factors that the current literature lacks.

## 2. Methods

We conducted two systematic reviews—a pre-clinical and a clinical systematic review. Both systematic reviews were performed according to the Preferred Reporting Items for Systematic Reviews and Meta-Analysis (PRISMA) guidelines [24].

### 2.1. Search Strategies

An a priori protocol based on the PRISMA-P checklist to perform a systematic literature search was prepared for both animal and clinical studies and uploaded to the International Prospective Register of Systematic Reviews (PROPSPERO; protocol ID: CRD42018091050 and CRD42018091793, respectively). PubMed and Embase were then searched with regards to their individual thesauruses. The following primary keyword query was applied: “Burns” AND (“MSC” OR “ASC”). For the complete search strategies for each database, please refer to Table 1.

### 2.2. Eligibility Criteria

To be valid for inclusion in the review, the studies were required to be either randomized or non-randomized controlled studies with full-text availability in English published between 2010 and January 2020. Moreover, the study populations were stipulated to be either rat/mouse dermal burn models or humans, all with thermal burn or scalding resulting in second- or third-degree burn wounds of partial or full thickness, or post-burn-wound excisional wounds. The intervention was primarily stem cell treatment using MSCs derived from the bone marrow, umbilical cord, dental pulp, or adipose tissue delivered as purified cells or fat grafts.

### 2.3. Study Selection and Data Extraction

For both reviews, the articles were imported into the Cochrane-partnered software Covidence (https://www.covidence.org/). Each study was screened by two independent reviewers based on title and abstract, after which a full-text screening was performed. Studies that did not meet the criteria were excluded. A bias assessment was performed using the Cochrane risk of bias (RoB) Tool SYRCLE [25] for the pre-clinical studies or the National Heart, Lung, and Blood Institute (NHLBI) study quality assessment tools [26] for the human studies. The data extracted from the full papers were population size and species, intervention type, origin, delivery and dose, comparison, and global outcome. Additionally, secondary outcome parameters—which included visual appearance, investigations, or measures of inflammation, proliferation or neovascularization, granulation and re-epithelialization, and remodeling/scarring—were extracted.

### 2.4. Assessment of Continuity of Evidence

To assess the continuity of evidence between pre-clinical and clinical studies, as well as among the clinical studies to justify the clinical translation, a cross-citation analysis was performed. To this end, the bibliographies of the included clinical studies were examined to identify which of the included pre-clinical and clinical studies had been cited.

## 3. Results

**Figure 1:**
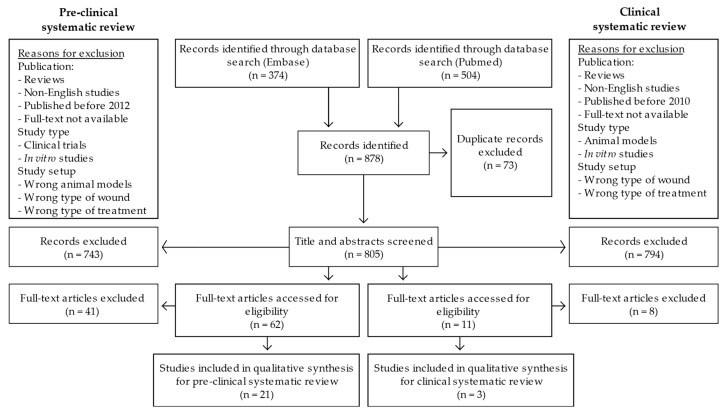
Preferred Reporting Items for Systematic Reviews and Meta-Analysis (PRISMA) flow diagram of the literature search and selection process. n, number.

The literature searches in Embase and Pubmed resulted initially in 374 and 504 publications, which, after removal of 73 duplicates, resulted in 805 unique records (Figure 1). These records were screened for eligibility for inclusion into either the pre-clinical review or the clinical review, first through screenings based on title and abstract, then through screening of full texts. The exclusion criteria were related to the publication, study type, and study setup (for details, please refer to Figure 1). The exclusion process resulted in a total of 21 pre-clinical and three clinical studies, respectively, which were included in the subsequent analyses (Figure 1).

### 3.1. Bias Assessment

For the pre-clinical studies, the bias assessment revealed that none of the studies reported any blinding of personnel or allocation concealment, and only 8 out of the 21 studies reported blinding of the outcome assessors (Table 2).

Regarding the randomization of the animals, all studies reported random outcome assessment, 15 studies reported random housing, and 12 studies published the method of randomization. The insufficient documentation as to how both blinding and randomization were performed is of concern, since these factors are central to accruing valid data.

The bias assessment of the human case studies (Table 3) highlighted the problematic nature of reporting on only a limited number of patients (one and two, respectively), resulting in a high risk of bias. The bias assessment of the case–control study Table 3) demonstrated a low risk of bias in the study.

In future randomized controlled clinical trials investigating the treatment of human burn victims with stem cells, a detailed description of blinding and randomization as well as the other bias types should be included. In order to increase the validity and reproducibility of the results, it would be beneficial if the bias assessment tools were already implemented in the design phase of the clinical trials. This would, in turn, increase the level of evidence in the produced results.

### 3.2. Study Characteristics

The 21 pre-clinical studies were published between 2013 and 2019 and the three clinical studies were published between 2012 and 2018. For both of the systematic reviews, the studies were analyzed in terms of population, intervention, comparison, global outcome (Table 4 and Table 5, respectively), and the effect of stem cells on the different phases of wound healing (Table 6 and Table 7, respectively).

#### 3.2.1. Population

The pre-clinical studies were all in vivo studies and the population size varied between 6 and 84 animals, with an average of 29.5 animals per study. The majority (15) of the studies were based on a rat model, and a mouse model was employed in six instances. The clinical studies consisted of one case–control study with 60 patients in total and two case reports with one or two patients.

#### 3.2.2. Intervention

For the analysis of the intervention used across studies, information about the type and origin of stem cells, delivery method, and dose was extracted.

Of the 21 pre-clinical studies, 10 used ASCs, which, in two cases, were in the form of stromal vascular fraction (SVF), seven studies used BM-MSCs, one study used ASCs, BM-MSCs, and dental pulp stem cells (DPSCs), and three studies used umbilical cord (UC)-MSCs. Stem cells from the same species were used in 12 studies, and human stem cells were used in nine. The method of delivery varied, with 14 studies using injection, six using graft, and one using spray. The doses used covered a broad range from 1 × 10^4^ to 6.8 × 10^6^ cells, with an average of 1.6 × 10^6^ cells.

For the clinical studies, one used ASCs in the form of SVF. The two other studies used BM-MSCs, and one additionally used UC-MSCs. All BM-MSCs were autologous, and the UC-MSCs were allogeneic. The method of delivery was either injection or topical administration. The doses used in the three different studies are incomparable, as they are stated in either cells/mL/cm^2^, cells/mL or cells/cm^2^. It is also notable that the treatment in the case–control study by Abo-Elkheir et al., 2017 [50] was repeated twice.

#### 3.2.3. Comparison

All the pre-clinical studies included a control group, a sham group (1 study), or both (2 studies). In four studies, the stem cells were used in combination with either aloe vera, human amniotic membrane (HAM), or small intestine submucosa (SIS), whereas in the remaining 17 studies, only the stem cells were administered.

The three clinical studies compared the effect of stem cell treatment either to a control group using the standard treatment of early excision and grafting, to a wound not receiving stem cells on the opposite arm of the included patient, or to the pre-intervention wound state. One study investigated the effect of stem cells together with a dressing with gentamicin ointment, one together with a decellularized allogeneic dermal matrix and a skin graft, and the last one together with an Integra template.

#### 3.2.4. Outcome

Overall, in all pre-clinical studies, the application of mesenchymal stem cells to the in vivo burn wound models had a positive effect (Table 4). A more detailed analysis of the effect of stem cells on the different phases of wound healing is presented in Table 6.

Of the 17 studies that assessed wound healing by either measuring the wound area or closure rate, 15 found a positive effect, one study did not show a significant effect, and one study documented no effect. The effect of stem cells on inflammation was evaluated in 12 studies. Of those, 10 demonstrated an anti-inflammatory effect and two studies were inconclusive. Of the 13 studies that evaluated the effect on neovascularization, 12 showed a positive effect and one was inconclusive. Regarding granulation, 13 studies demonstrated a positive effect and one study showed no effect. As for re-epithelialization, 11 studies demonstrated a positive effect and one study showed no effect. The effect of stem cells on remodeling and scarring was investigated in eight studies; five of these demonstrated a positive effect, two were inconclusive, and one reported no difference.

All three clinical studies found a positive effect of the application of mesenchymal stem cells to the burn wounds. However, in the case–control study by Abo-Elkheir et al. (2017), infection was observed in 25–70% of patients receiving stem cell treatment compared to 25% in the control group (Table 7).

Xu et al. (2012) found no difference in the overall healing, as both wounds in the trial subject were healed when progress was investigated. Moreover, no difference in inflammation was observed, as none of the wounds became infected. All three studies found a positive effect on scarring with a noticeable decrease in contracture.

### 3.3. Outcome Assessment Parameters

For each of the studies, the phases of the wound healing were further characterized by invoking additional outcome assessment parameters (Table 8 and Table 9). For the animal studies, a range of both non-invasive and invasive methods were applied. However, in the case of clinical studies, only visual assessment was used for the evaluation of overall wound healing and for the evaluation of the effect of stem cells on the different phases (Table 9). 

#### 3.3.1. Wound Healing

Non-invasive assessment of overall wound healing was used in 17 of 21 studies in the form of either end-point measurements or rate of wound closure. Some studies determined the necrotic area as a measure of tissue damage. In addition to the non-invasive assessment, four studies applied a wound healing or maturation scoring system based on histological data.

#### 3.3.2. Inflammation

Non-invasive assessment of inflammation or infection was used in three studies. The assessments included signs of inflammation, such as redness, heat, and swelling, and the bacterial contamination was determined by swabbing. Biopsies were used for histological assessment of the infiltration by inflammatory cells (11 studies), and quantitation of expression of interleukin-1b (*IL-1b*), transforming growth factor beta 1 (*TGF-β1*), basic fibroblast growth factor (*bFGF*), macrophage-inflammatory protein 2 (*MIP2*), or tumor necrosis factor-alpha 1 (*TNFα1*) was assessed using quantitative polymerase chain reaction (qPCR) (two studies), myeloperoxidase activity (two studies), and flow cytometry of the CD4+ and CD8+ T cells (one study). Three studies used blood samples to determine white blood cell count or c-reactive protein (CRP) levels, or used enzyme-linked immunosorbent assay (ELISA) to quantitate levels of IL-6, IL-10, TGF-β, cytokine-induced neutrophil chemoattractant (CINC-1), TNF-α, and interferon gamma (IFN-γ).

#### 3.3.3. Proliferation

Our data analysis of the methods used to analyze the proliferative phase of wound healing divides them further into three processes—neovascularization, granulation, and re-epithelialization.

##### Neovascularization

Most of the studies investigated neovascularization based on biopsies. The methods included histomorphometry to determine capillary density (six studies), immunohistochemistry to investigate the presence of platelet-derived growth factor (PDGF), VEGF, vascular endothelial growth factor receptor 2 (VEGFR2), or isolectin B4 (IB4) as a measure of neovascularization, as well as the presence of von Willebrand factor (vWF) or platelet endothelial cell adhesion molecule (CD31) to estimate the number of endothelial cells (nine studies). Furthermore, qPCR was used to study the expression of *IL-1b*, *bFGF*, *VEGF-α1*, *VEGFR2*, angiopoietin 1 and 2 (*Ang-1/2*), or *CD31* (three studies), one study used western blotting (WB) to determine the levels of VEGF, Ang-1/2, and CD31, and, finally, in one study, blood samples were tested for TGF- β using ELISA.

##### Granulation

The process of granulation was, in most studies, evaluated in biopsy samples. Histological analysis was used to determine the degree of cell proliferation (three studies) and the presence of fibroblasts and/or fibrocytes (two studies), as well as to characterize the ECM in terms of the amount and composition of collagens (seven studies) and integrins (one study). In one study, the ECM was analyzed by scanning electron microscopy. The transcriptional activation of collagen I, collagen III, or vimentin genes was determined by qPCR in three studies. WB was used to assess the amount of collagen I, collagen III, TGF-β, or VEGF in two studies, and, lastly, a serum-based ELISA was used in one study to measure the level of TGF-β.

##### Re-Epithelialization

In all 12 studies that specifically dealt with the process of re-epithelialization, the evaluation was done using biopsies. Eleven studies used histological analysis to determine the epidermal formation, epidermal cell proliferation, epidermal thickness, hair follicle formation, rete-ridges, and dermal appendages. qPCR was used in two studies to either investigate wound contraction by alpha smooth muscle actin (*α-SMA*) or to investigate metalloproteinases and their inhibitors: matrix metalloproteinase-1 (*MMP-1*) and tissue inhibitor of metalloproteinases 2 (*TIMP-2*). One study used a serum-based ELISA to measure the level of TGF-β.

#### 3.3.4. Remodeling/Scarring

Of the 21 studies, only seven investigated the processes of remodeling and scarring. Of these, six were based on biopsies and included histological assessment of connective tissue arrangement or pathologic dermal fibrosis. Immunohistochemistry was used in one study to quantitatively evaluate scar formation by means of visualization of collagens I, III, and IV. Additionally, qPCR was used in two studies to determine the transcriptional levels of collagens I, II, and IV, *MIP-2*, *TGF-β1*, *TNF-α1*, and *MMP-1* and *-2*. Furthermore, in one study, ELISA was used to assay the serum level of TGF-β.

### 3.4. Pre-Clinical to Clinical Continuity of Evidence

When performing a cross-citation analysis to assess the evidence used to justify clinical translation, none of the pre-clinical studies were identified in the bibliographies of any of the clinical studies. Moreover, none of the clinical studies cited the other clinical studies.

## 4. Discussion

In the current systematic review, we have evaluated the research of therapeutic use of stem cells towards healing of burn wounds. A total of 21 pre-clinical and three clinical studies were included. The pre-clinical studies investigated wound healing by looking at different micro-processes of the different wound healing phases, such as inflammation, proliferation, neovascularization, granulation, re-epithelialization, and remodeling/scarring. Noticeably, the studies varied widely due to differences in scientific focus, research strategy, and methods used; therefore, it was not possible to directly compare their findings. Nevertheless, there was apparent positive effect of MSCs on the overall healing, and the majority of studies agreed on the positive effect on all of the individual phases as well (Table 5). From the temporal viewpoint, most of the studies investigated the progressive effect of MSCs, typically 7, 14, and 21/28 days after burn infliction; however, some studies also looked at the effect after 40–60 days. The effect was most evident between 7 and 21 days, at which point 90% of the wounds were healed. It should be noted that a rapid wound closure is of importance to decrease the risk of hypermetabolism, infection, and hypovolemic shock [1].

In order to identify the wound healing processes where the MSCs exerted their regenerative properties, the effect on each phase and the related specific micro-processes were examined. For the inflammation phase, 10 out of 12 studies reported immunomodulatory effects. For neovascularization, 12 out of 14 studies found a positive effect. Effects were also found in 13 out of 14 studies in regard to the granulation phase, as well as 11 out of 12 studies for the re-epithelialization phase. Only eight studies examined the effect of treatment on the remodeling/scarring phase. Out of these, five reported a positive effect, which was, incidentally, associated with the use of ASCs. That ASCs may be specifically useful in this stage, where extensive ECM remodeling takes place, also gains support from an in vitro line of evidence [15]. In burn wounds, the importance of this phase cannot be overstated, as scarring, soft tissue deformities, and fibrosis represent serious long-term consequences [2].

To evaluate the mechanism of action of MSCs in the healing of burn wounds, a frequently used approach was the histological assessment. However, due to the non-quantitative nature of this procedure, it was difficult to arrive at evidence-based conclusions. Some of the studies converted their findings into semi-quantitative scoring systems to enable a sort of comparison. In the face of obvious deficiency of such attempts, it would be of great value if a unified scoring system was developed and adopted across the wound healing research community. It is important that future studies strive to incorporate quantitative assays in their experimental design as much as possible, and that these approaches are applied in a way reflecting wound pathogenesis. As an example, we have identified several studies that attempted to determine the role of bFGF or TGF-β. Despite providing information about overall production, they were not able to highlight an association of these factors with a particular phase of healing. This could have been useful, since such information would facilitate a more in-depth understanding of the MSCs’ interaction with the wound microenvironment. Future animal studies should also apply tools like SYRCLE in the design stage. This, including the minimization of risk of bias, should clearly be spelled out in the published works. Moreover, new studies should be designed to be confirmatory studies, as these are increasingly considered to be the necessary prerequisite for clinical trials [51,52].

Although a substantial number of studies involving MSC application have been conducted in animal models, very few studies have been investigating the effect in humans. These studies have yielded a very low level of evidence due to the study type and very limited outcome measures. To take MSCs into consideration as a standard treatment option, the amount of evidence needs to be considerably increased. To this end, randomized controlled trials need to be designed with emphasis on risk of bias, sample size, and choice of primary and secondary outcome measures. Additionally, it is important that the knowledge gap is addressed, and the available clinical and pre-clinical evidence is used in the rationalization step. When undertaking these studies, it is encouraged to thoroughly consider the risk areas, such as the randomization process, deviations from intended interventions, missing outcome data, measurement of the outcome, or selection of the reported results, which can all lead to high risk of bias in randomized controlled trials. These areas can be found through risk assessment tools, such as the Cochrane risk of bias tool (RoB2) [53]. Another important factor, that should be given thorough consideration, is the sample size, since this variable is critical for the outcome of statistical analysis in terms of significance and power. However, as a large study population can be difficult to muster, due to the incidence of burn wounds, it should be remembered that sample size and covariates must be balanced, especially in smaller clinical trials [54,55].

It appears that the most meaningful progress in understanding the clinical utility of MSCs would occur if the primary and secondary outcome measures included quantitative analysis of the biopsy samples for investigating specific markers for each of the wound healing phases. Histological analysis of the biopsies could include quantitation of infiltrating inflammatory cells, proliferating endothelial cells or fibroblasts, or collagen composition to assess progression through the inflammation, proliferation, or remodeling phases, respectively. However, ethical concerns regarding inflicting further trauma make it difficult to collect biopsies from these complex wounds. In case the justification is serious enough to warrant the sampling, it must be guaranteed that the utilization of the material is maximized. Therefore, a well-designed panel of quantitative assays should be conducted to clarify MOA. In cases where more than one biopsy during the healing period is needed, the timing of these should be designed to specifically target the different wound healing phases. Based on the animal studies reviewed in this paper, we suggest days 7, 14, and 21, and perhaps a biopsy after a year to monitor the long-term wound maturation.

The presented evidence indicates that the stem cell treatment is generally beneficial, although it seems that the animal model is not entirely identical to the human scenario. Notably, we observed that whereas the MSCs appeared effective throughout all phases of wound healing in the pre-clinical trials, their effect was mostly reduced to modulation of scarring and contracture in patients. Moreover, we noted an obvious lack of coherence in knowledge translation from bench to bedside, and the number of clinical studies was disappointingly low and had a very low evidence level with a high risk of bias. Thus, more clinical studies are needed, and, hopefully, identifying and recognizing the knowledge gaps and the discontinuity from pre-clinical to clinical research may promote better design of future clinical studies.

In summation, this article, based on two systematic reviews, provides a comprehensive overview of the available methodologies and MOA of MSC treatment of burn wounds from both pre-clinical and clinical studies. Although the animal trials produced some exciting results, it is evident that high-quality randomized controlled trials are required before drawing universal conclusions about the clinical effect of stem cell treatment in burn wounds.

## Figures and Tables

**Table 1:** Database search strategy.

Database	Search Strategy
**PubMed**	(Burn [MeSH] OR “Thermal injury” OR “second degree burn” OR “third degree burn” OR “skin burn” OR “burn trauma” OR “burn damage” OR “burn wound” OR “burn injur *”OR “burn patient *” OR “burn lesion” OR “burn complication” OR “deep burn” OR “thermal burn” OR “deep partial thickness” OR “full thickness”) AND (Mesenchymal stromal cells [MeSH] OR “Adipose mesenchymal stem cell *” OR “adipose derived mesenchymal cell *”) AND (“Adipose stromal cell *” OR “Adipose tissue-derived stem cell *” OR “adipose-derived adult stem cell *” OR “adipose derived regenerative cell *”)
**Embase**	(Exp/burn OR Exp/thermal injury) AND (Exp/mesenchymal stem cell OR Exp/mesenchymal stromal cell OR “Adipose mesenchymal stem cell *”, “adipose derived mesenchymal cell *”) OR (Exp/adipose derived stem cell OR (“adipose derived stem cell” OR “adipose stromal cell *” OR adipose tissue-derived stem cell *” OR “adipose-derived adult stem cell *” OR “adipose derived regenerative cell *”)

* Unlimited truncation.

**Table 2:** A visual representation of the risk of bias in animal studies.

	Sequence Generation	Allocation Concealment	Blinding of Personnel	Blinding of Personnel Assessors	Incomplete Outcome Data	Selective Reporting	Other Bias	Random Housing	Baseline Characteristics	Random Outcome Assessment
Oryan et al. [27]										
Atalay et al. [28]										
Eyuboglu et al. [29]										
Chen et al. [30]										
Loder et al. [31]										
Shokrgazor et al. [32]										
Kaita et al. [33]										
Bliley et al. [34]										
Gholipourmalekabadi et al. [35]										
Motamed et al. [36]										
Abbas et al. [37]										
Caliari-Oliveira et al. [38]										
Ahmed et al. [39]										
Singer et al. [40]										
Revilla et al. [41]										
Oh et al. [42]										
Guo et al. [43]										
Xue et al. [44]										
Zhang et al. [45]										
Pourfath et al. [46]										
Gholipour-Kanani et al. [47]										

Red: high risk of bias; green: low risk of bias.

**Table 3:** A visual representation of the risk of bias in human case series and case-control studies.

(**A**) A visual representation of the risk of bias in human case series studies.												
	**Study Question/Objective**		**Study Population**	**Consecutive Cases**	**Comparable Subjects**	**Description of Intervention**	**Outcome Measures**	**Follow-Up Length**	**Statistical Analysis**	**Well Described Results**		
Xu et al., 2012 [48]												
Arkilous et al., 2018 [49]												
(**B**) A visual representation of the risk of bias in human case–control studies.												
	**Objective**	**Study Population**	**Sample Size Justification**	**Controls**	**Use of Inclusion/Exclusion Criteria**	**Case Definition**	**Random Selection**	**Concurrent Controls**	**Exposure**	**Exposure Measurement**	**Blinding**	**Statistical Analysis**
Abo-Elkheir et al., 2017 [50]							NA		NA		NA	

Red: high risk of bias; green: low risk of bias. NA, not applicable.

**Table 4:** Summary of populations, interventions, comparisons, and global outcomes for pre-clinical studies.

Study	Population		Intervention				Comparison	Outcome (Global)
	Sample Size	Species	Type	Origin	Delivery	Dose		
Oryan et al., 2019 [27]	12	R	ASC	Murine	Injection	1 × 10^6^	ASC + Aloe Vera Aloe Vera Aloe Vera + DBM DBM	Positive, significantly better than other groups
Atalay et al., 2014 [28]	20	R	ASC *	Murine	Injection	4 × 10^6^	ASC Control	Positive
Eyuboglu et al., 2018 [29]	20	R	ASC *	Murine	Injection	4 × 10^6^	ASC Control	Positive
Chen et al., 2017 [30]	6	R	ASC	Murine	Injection	1 × 10^6^	ASC Control	Positive
Loder et al., 2015 [31]	20	M	ASC	Murine	Injection	1 × 10^6^	ASC AT AT + ASC Sham	Positive, significantly better than non-stem cell groups
Shokrgazor et al., 2012 [32]	10	R	ASC	Murine	Graft	5 × 10^5^	ASC Control	Positive
Kaita et al., 2019 [33]	18	M	ASC	Human	Graft	5 × 10^4^	Fresh Frozen Control	Positive
Bliley et al., 2016 [34]	24	M	ASC	Human	Injection	6.8 × 10^6^	ASC Control	Positive, but limited
Gholipourmalekabadi et al., 2018 [35]	45	M	ASC	Human	Graft	1 × 10^4^	HAM HAM + ASC Control	Positive. More significant in HAM + ASC
Motamed et al., 2017 [36]	24	R	ASC	Human	Graft	5 × 10^5^	HAM HAM + ASC Control	Positive. More significant in HAM + ASC
Abbas et al., 2018 [37]	40	R	ASC, BM-MSC, DPSC	Human	Injection	1 × 10^6^	BM-MSC ASC DPSC Control	Positive, no difference between choice of stem cells
Caliari-Oliveira et al., 2016 [38]	54	R	BM-MSC	Murine	Injection	5 × 10^6^	MSC Control	Positive
Ahmed et al., 2017 [39]	36	R	BM-MSC	Murine	Injection	1 × 10^6^	MSC Control Sham	Positive
Singer et al., 2013 [40]	20	R	BM-MSC	Murine	Injection	1 × 10^6^	MSC Control	Positive
Revilla et al., 2016 [41]	12	R	BM-MSC	Murine	Injection	2 × 10^6^	MSC Control	Positive
Oh et al., 2018 [42]	30	M	BM-MSC	Murine	Injection	5 × 10^5^	MSC Control Sham	Positive
Guo et al., 2016 [43]	48	R	BM-MSC	Murine	Graft	5 × 10^5^	SIS SIS + MSC Control	Positive. More significant in SIS + MSC
Xue et al., 2013 [44]	60	M	BM-MSC	Human	Injection	1 × 10^6^	MSC Control	Positive
Zhang et al., 2015 [45]	84	R	UC-MSC	Human	Injection	2 × 10^6^	MSC Control	Positive
Pourfath et al., 2018 [46]	24	R	UC-MSC	Human	Spray	5 × 10^5^	MSC Control	Positive
Gholipour-Kanani et al., 2014 [47]	12	R	UC-MSC	Human	Graft	4 × 10^4^	MSC Control	Positive

ASC: adipose-derived stem cells, AT: adipose tissue, BM-MSC: bone-marrow-derived mesenchymal stem cells, DBM: demineralized bone matrix, DPSC: dental pulp stem cells, HAM: human amniotic membrane, M: mouse, MSC: mesenchymal stem cells, R: rat, SIS: small intestine submucosa, UC-MSC: umbilical cord mesenchymal stem cells. * ASCs delivered as stromal vascular fraction.

**Table 5:** Summary of populations, interventions, comparisons, and global outcomes for clinical studies.

Study	Population		Intervention				Comparison			Outcome (Global)
	Sample Size	Species	Type	Origin	Delivery	Dose				
Xu et al., 2012 [48]	1	H	BM-MSC	Autologous	Injection	2.1 × 10^6^/mL	BM-MSC + Decellularized allogeneic dermal matrix + Skin graft	Decellularized allogeneic dermal matrix + Skin graft		Positive
Arkoulis et al., 2018 [49]	2	H	ASC *	Autologous	Topical	46,400 /cm^2^	Pre-intervention	Post-intervention		Positive
Abo-Elkheir et al., 2017 [50]	60	H	BM-MSC	Autologous	Injection	1 × 10^5^/mL/cm^2^ × 2	BM-MSC + dressing with gentamicinointment	UC-MSC + dressing with gentamicinointment	Standard treatment	Positive
			UC-MSC	Allogeneic						

ASC: adipose-derived stem cells, BM-MSC: bone-marrow-derived mesenchymal stem cells, H: human, UC-MSC: umbilical cord mesenchymal stem cells. * ASCs delivered as stromal vascular fraction.

**Table 6:** Analysis of the outcomes according to the phases of wound healing in pre-clinical trials.

	Wound Healing	Inflammation	Proliferation			Remodeling/Scarring
			Neovascularization	Granulation	Re-Epithelialization	
Oryan et al., 2019 [27]						
Atalay et al., 2014 [28]						
Eyuboglu et al., 2018 [29]						
Chen et al., 2017 [30]						
Loder et al., 2015 [31]						
Shokrgazor et al., 2012 [32]						
Kaita et al., 2019 [33]						
Bliley et al., 2016 [34]						
Gholipourmalekabadi et al., 2018 [35]						
Motamed et al., 2017 [36]						
Abbas et al., 2018 [37]						
Caliari-Oliveira et al., 2016 [38]						
Ahmed et al., 2017 [39]						
Singer et al., 2013 [40]						
Revilla et al., 2016 [41]						
Oh et al., 2018 [42]						
Guo et al., 2016 [43]						
Xue et al., 2013 [44]						
Zhang et al., 2015 [45]						
Pourfath et al., 2018 [46]						
Gholipour-Kanani et al., 2014 [47]						

Green: Positive effect; red: non-significant difference or contradictory results; yellow: no difference; grey: not assessed.

**Table 7:** Analysis of the outcomes according to the phases of wound healing in clinical trials.

	Wound Healing	Inflammation	Proliferation	Remodeling/Scarring
Xu et al., 2012 [48]				
Arkoulis et al., 2018 [49]				
Abo-Elkheir et al., 2017 [50]				

Green: Positive effect; red: non-significant difference or contradictory results; grey: not assessed.

**Table 8:** Outcome assessment parameters from pre-clinical trials.

Study	Wound Healing	Inflammation	Proliferation			Remodeling/Scarring
			Neovascularization	Granulation	Re-Epithelialization	
Oryan et al., 2019 [27]	Wound area, Rate of wound closure (NI)	Inflammation markers (visual inspection, NI). Inflammatory cell infiltration (H, BI). IL-1b, TGF-β1, bFGF (qPCR, BI)	Capillary density (H, BI)	Collagen structure (SEM, BI). Number of fibroblasts and fibrocytes (H, BI). Collagen level (Hydroxyproline, BI)	Epidermal formation (H, BI)	Connective tissue arrangement (H, BI)
Atalay et al., 2014 [28]		Polymorphonuclear and mononuclear inflammatory infiltrate score (H, BI)	VEGF index (VEGF; H + I, BI)	Cell proliferation index (PCNA; H + I, BI)		
Eyuboglu et al., 2018 [29]	Area of necrosis (NI)	Neutrophil score (H, BI)	Capillary count (Angiography + H, BI). Vascular density grading (H, BI). Endothelial count (vWF; H + I, BI)		Epithelial thickness (H, BI)	Fibrosis gradient (Masson’s trichrome, BI)
Chen et al., 2017 [30]	Wound area, Rate of wound closure (NI)	Lymphocytic inflammatory infiltration (H, BI)			Epithelial regeneration (H, BI)	Pathologic dermal fibrosis (H, BI)
Loder et al., 2015 [31]	Wound area closure (NI). Wound depth, Rate of wound closure (BI)		Endothelial count (CD-31; H + I, BI)	Proliferation (Ki67; H + I, BI)		
Shokrgazor et al., 2012 [32]	Wound area (NI)				Epidermal formation (H, BI)	
Kaita et al., 2019 [33]	Wound area, Rate of wound closure (NI)		Neovascularization (IB4; I, BI)	Collagen production (Picro-Sirus Red, Col I/III; H + WB + qPCR, BI)	Skin thickness ratio (Masson’s trichrome, BI)	
Bliley et al., 2016 [34]	Wound area, Rate of wound closure, Wound area, Time to healing (NI)		Vascularity (CD31; H + I, BI)	Collagen production (Picro-Sirius Red, Masson’s trichrome; H) (Col I, Col III; qPCR, BI)	Wound contraction (α-SMA; qPCR, BI)	Collagen production (Col I and III; qPCR, BI)
Gholipourmalekabadi et al., 2018 [35]	Wound area, Rate of wound closure, Wound area (NI). Wound-healing scoring (H, BI)	Acute inflammatory cells (H, BI). Localized Inflammatory Response (MIP2, TNFα1, and TGFβ1; qPCR, BI)	Capillary density (CD31; I, BI). Neovascularization score (CD31, VEGF- α1, VEGFR2; I, BI). Neovascularization rate (IL-1b, bFGF, VEGF-α1, VEGFR2; qPCR, BI)	Deposition of the extracellular matrix (H, BI). Collagen deposition score (Masson’s trichrome, BI). Density of Col I, III, and IV (I, BI).	Hair follicle formation (H, BI). Re-epithelialization (H, BI), Epidermal Thickness Index.	Maturation (Masson’s trichrome, BI). Scar formation (Col I, III, and IV; I) (Col I, III, IV, MIP-2, TGFβ1, TNFα1, MMP-1, MMP-2; qPCR, BI). Scar Elevation Index.
Motamed et al., 2017 [36]	Wound area, Rate of wound closure (NI)	Acute inflammatory cells (polymorphonuclear cells, eosinophils; H + Masson’s trichrome, BI), Chronic inflammatory cells (histocytes, lymphocytes, plasma cells; H + Masson’s trichrome, BI)			Epidermal and dermal structures, re-epithelialization, epithelium thickness, rete-ridges, dermal appendages (H + Masson’s trichrome, BI)	Pathologic dermal fibrosis (H + Masson’s trichrome, BI)
Abbas et al., 2018 [37]	Area of necrosis (NI)	Inflammatory Cell Infiltration (myeloperoxidase activity, BI)	Microvascular density (CD31; H + I, BI)			
Caliari-Oliveira et al., 2016 [38]	Wound area Rate of wound closure (NI)	Bacterial contamination (swabs, NI). Total polymorphonuclear inflammatory cells score (H, BI). Neutrophils accumulation (myeloperoxidase assay, BI). CD4+ T-cells and CD8+ T-cells (Flow, BI). IL-10, IL-6, TGF-β, CINC-1 (ELISA, BS)	Vascularization score (H, BI)	Granulation tissue thickness score (H, BI)		Collagen fiber score (H, BI)
Ahmed et al., 2017 [39]		Acute inflammatory cells (H, BI). IL-10, TNF-α, TGF-β (ELISA, BS)	Capillaries (H, BI), TGF-β (ELISA, BS). PDGF (I, BI). ANG-1, ANG-2 (qPCR, BI)	TGF-β (ELISA, BS). PDGF (I, BI). Vimentin (qPCR, BI)	Epithelialization (H, BS), TGF-β (ELISA, BS). MMP-1, TIMP-2 (qPCR, BI)	TGF-β (ELISA, BS)
Singer et al., 2013 [40]	Area of necrosis (NI)					
Revilla et al., 2016 [41]	Wound appearance (NI)			Collagen type I fiber thickness, Integrin a2b1 (H + I, BI)		
Oh et al., 2018 [42]		Inflammatory cell infiltration (Masson’s trichrome, BI)		Collagen production (Masson’s trichrome, BI). TGF-β1 and VEGF (WB, BI)		
Guo et al., 2016 [43]	Wound area, Rate of wound healing (NI). Wound maturity score (H, BI)		Capillary density (vWF; I, BI)	Granulation score (Collagen; Masson’s trichrome, BI)	Neoepithelium length (H, BI). Epidermal cell proliferation (Ki-67; I, BI)	
Xue et al., 2013 [44]	Wound area, Rate of wound healing (NI)		Capillary density (H, BI). VEGF, Ang-1/2, CD31 (qPCR, WB, BI).			
Zhang et al., 2015 [45]	Wound healing rate and time (NI)	WBC (count, BS). CRP (nephelometric immunoassay method, BS). IFN-γ, TNF-α, IL-6, IL-10 (ELISA, BS)	Capillary density (H, BI)	Granulation tissue amount (H, BI). Number of fibroblasts (H, BI)		
Pourfath et al., 2018 [46]				Granulation (H, BI)	Re-epithelialization (H, BI)	
Gholipour-Kanani et al., 2014 [47]	Wound area (NI). Total wound healing score (H, BI)	Inflammation markers (visual inspection, NI). Inflammatory cell infiltration (H, BI)		Collagen regeneration and granulation tissue thickness (H, BI)	Epithelial regeneration, appendage (H, BI)	

ANG-1/2: Angiopoietin-1/2, α-SMA: alpha smooth muscle actin, bFGF: basis fibroblast growth factor, BI: biopsy, BS: blood sample, CD-31: platelet endothelial cell adhesion molecule, CINC-1; cytokine-induced neutrophil chemoattractant, Col: collagen, CRP: C-reactive protein, ECM: extracellular matrix, ELISA: enzyme-linked immunosorbent assay, H: histology, I: immunohistochemistry, IFN-γ: interferon gamma, IL: interleukin, IB4: isolectin B4, Flow: flowcytometry, MIP-2: macrophage-inflammatory protein 2, MMP: matrix metalloproteinase, NI: non-invasive, PCNA: proliferating cell nuclear antigen, PDGF: platelet-derived growth factor, qPCR: quantitative polymerase chain reaction, SEM: scanning electron microscopy, TGF-β: transforming growth factor beta, TIMP-2: tissue inhibitor of metalloproteinases 2, TNFα: tumor necrosis factor alpha, VEGF: vascular endothelial growth factor, VEGFR2: vascular endothelial growth factor receptor 2, vWF: von Willebrand factor, WB: western blot, WBC: white blood count.

**Table 9:** Outcome assessment parameters from clinical trials.

Study	Wound Healing	Inflammation	Proliferation	Remodeling/Scarring
Xu et al., 2012 [48]	Overall healing (visual)	Infection (visual)		Contracture (visual)
Arkoulis et al., 2018 [49]				Contracture (visual)
Abo-Elkheir et al., 2017 [50]	Type of burn Onset, cause, mechanism, site, wound percentage (Lund and Browder), area of wound, depth of wound, rate of healing	Infection (visual)		Hypertrophic scars, keloid, contracture, and pigmentation (visual)
